# Use of Medical Care during Pandemic (H1N1) 2009, Navarre, Spain

**DOI:** 10.3201/eid1708.101817

**Published:** 2011-08

**Authors:** Rosana Burgui, Iván Martínez-Baz, Marcela Guevara, Silvia Carlos, Jesús Castilla

**Affiliations:** Author affiliations: Instituto de Salud Pública de Navarra, Pamplona, Spain (R. Burgui, I. Martínez-Baz, M. Guevara, J. Castilla);; Centro de Investigación Biomédica en Red de Epidemiología y Salud Pública, Pamplona (R. Burgui, I. Martínez-Baz, M. Guevara, J. Castilla);; Universidad de Navarra, Pamplona (S. Carlos)

**Keywords:** pandemic (H1N1) 2009, influenza, influenza-like illness, viruses, epidemiology, medical care, Spain, dispatch

## Abstract

Of 233 households with laboratory-confirmed pandemic (H1N1) 2009 in Navarre, Spain, only 64% (107/166) of contacts with influenza-like illness had sought medical care. This value was lower for adults (53%, 39/74) than for children <15 years of age (74%, 68/92), as well as for those with cases secondary to another household case (58%, 64/111).

Every year, influenza affects 5%–10% of the population, and most persons have various episodes of this disease during their lifetime (*1**,**2*). Influenza is typically characterized by respiratory symptoms and general symptoms such as fever, malaise, and myalgia (*2**,**3*). Most cases resolve without complications within 1 week (*4*). The epidemiologic context facilitates diagnosis of influenza because it usually occurs in seasonal waves (*5*). For these reasons, some persons with influenza do not seek medical care.

This proportion of influenza cases is hidden from the health system and epidemiologic surveillance, leading to underestimation of the true extent of the affected population. The purpose of this study was to estimate the proportion of persons with influenza who sought medical care in Navarre, Spain, during the 2009–10 influenza season and identify characteristics that differentiate persons who sought or did not seek medical care.

## The Study

The study protocol was reviewed and approved by the Ethical Committee of the Barcelona Health Institute. During the 2009–10 influenza season, the Sentinel Network of Primary Care Physicians and Pediatricians of Navarre obtained nasopharyngeal swab specimens from patients with influenza-like illness (ILI) for virologic confirmation of influenza by using standard real-time reverse transcription PCR or cell culture (*6*).

In early 2010, a trained nurse telephoned households of persons who had had laboratory-confirmed pandemic (H1N1) 2009 in October and November 2009 while pandemic influenza was active in the area (index cases). When no response was received, the calls were repeated 5 times on different days and at different times. The interview was conducted by using a structured questionnaire. For each household, an attempt was made to talk to the adult who was primarily responsible for the health issues of those who lived there, usually the mother or father. When possible, other adults in the household were also interviewed.

Detailed information was obtained about index case-patients and all other persons living in the same household (contacts) with regard to sociodemographic data, medical history, influenza symptoms, and whether he or she had sought medical care. A question was asked about each of the following signs and symptoms: fever, cough, sore throat, headache, muscular or joint pain, nasal congestion, and vomiting.

This analysis included all household residents who had ILI for <7 days with respect to the onset of ILI in the index case-patient. ILI was defined as fever and either cough or sore throat in the absence of other diagnoses. The first person with ILI in the household was considered the primary case-patient, and others, if any, were considered secondary case-patients. Persons with index cases who led us to contact the household were excluded from analysis because, given the study design, they had all sought medical care.

Of 252 households that met the inclusion criteria, 233 (92%) were successfully contacted, and all persons agreed to participate in the study. Of 668 household contacts of index cases, 188 (28%) persons had >1 influenza symptoms and 166 (25%) met the criteria for having ILI. The proportion of symptomatic cases that met the criteria for ILI was higher among children (94%, 92/98) than among adolescents and adults (82%, 74/90; p = 0.021).

Of 166 persons with ILI, only 107 (64%, 95% confidence interval [CI] 57%–72%) had sought medical care. The percentage of those who sought medical care was lower among adults and adolescents (53%, 39/74) than among children <15 years of age (74%, 68/92; p = 0.006). This percentage was also lower among persons with cases secondary to a previous household case (58%, 64/111) than among primary case-patients (78%, 43/65; p = 0.010) (Table 1).

**Table 1:** Persons with ILI who sought medical care, by sociodemographic and health-related characteristics, Navarre, Spain, 2009–10*

Characteristic	No. persons with ILI	No. (%) who sought medical care	Crude OR (95% CI)	p value†
Sex				
M	86	55 (64)	1	
F	80	52 (65)	1.0 (0.6–2.0)	1.000
Age, y				
<15	92	68 (74)	2.5 (1.3–5.2)	0.006
>15	74	39 (53)	1	
Residence				
Rural	45	28 (62)	1	
Urban	121	79 (65)	1.1 (0.6–2.3)	0.719
Country of origin				
Spain	146	95 (65)	1	
Other	20	12 (60)	0.8 (0.3–2.1)	0.804
Major chronic conditions				
No	146	93 (64)	1	
Yes	20	14 (70)	1.3 (0.5–3.7)	0.629
Seasonal influenza vaccine				
No	149	94 (63)	1	
Yes	17	13 (76)	1.9 (0.6–6.1)	0.423
Smoked				
No	147	98 (67)	1	
Yes	19	9 (47)	0.5 (0.2–1.2)	0.127
Relation to other cases in household				
Secondary case	111	64 (58)	1	
Primary case	55	43 (78)	2.6 (1.3–5.5)	0.010
Antiviral treatment for primary case-patient				
No	75	45 (60)	1	
Yes	36	19 (53)	0.7 (0.3–1.7)	0.540
Total	166	120 (64)		

*ILI, influenza-like illness; OR, odds ratio; CI, confidence interval. †By Fisher exact test.

No differences were detected by sex, rural/urban residence, country of origin, vaccination against seasonal influenza, smoking status, or presence of major chronic conditions. The percentage of secondary case-patients who sought medical care was the same regardless of whether the primary case-patient received antiviral treatment. Persons who had sought medical care had a mean ± SD of 4.0 ± 0.8 symptoms, which was similar to those who had not sought medical care (4.0 ± 0.7 symptoms; p = 0.688). None of the contacts had received antiviral prophylaxis or vaccine for pandemic (H1N1) 2009.

The frequency with which persons with ILI sought medical care, by age group and symptoms, is shown in Table 2. Frequency of symptoms did not differ between persons who had sought medical care and those who had not sought medical care.

**Table 2:** Persons with ILI who sought medical care, by age and signs or symptoms, Navarre, Spain, 2009–10*

Sign or symptom	Children <15 y of age				Adolescents and adults		
	No. persons with ILI	No. (%) who sought medical care	p value†		No. persons with ILI	No. (%) who sought medical care	p value†
Fever			ND				ND
No	0	0			0	0	
Yes	92	68 (74)			74	39 (53)	
Cough			1.000				1.000
No	4	3 (75)			7	4 (57)	
Yes	88	66 (75)			67	35 (52)	
Sore throat			0.464				0.815
No	35	24 (69)			42	23 (55)	
Yes	57	44 (77)			32	16 (50)	
Headache			0.342				0.759
No	44	35 (80)			12	7 (58)	
Yes	48	33 (69)			62	32 (52)	
Muscle pain			0.180				0.241
No	86	65 (76)			32	14 (44)	
Yes	6	3 (50)			42	25 (60)	
Rhinorrhea			0.804				0.479
No	29	21 (72)			44	25 (57)	
Yes	63	47 (75)			30	14 (47)	
Vomiting			0.277				0.473
No	81	58 (72)			73	39 (53)	
Yes	11	10 (91)			1	0	

*ILI, influenza-like illness; ND, not determined. †By Fisher exact test.

Logistic regression analysis showed that seeking medical care was more frequent among children than among adolescents and adults (adjusted odds ratio 2.2, 95% CI 1.1–4.3; p = 0.019) and among primary case-patients than among secondary case-patients (adjusted odds ratio 2.2, 95% CI 1.0–4.8; p = 0.038). A similar proportion of contacts with any symptom but who did not meet ILI criteria sought medical care (59%, 13/22) as did those who met ILI criteria (64%, 107/166; p = 0.642).

## Conclusions

We studied persons who had ILI and were household contacts of persons with laboratory-confirmed influenza. It is likely that the cause of symptoms among persons with ILI was also infection with influenza virus. Approximately two thirds of these persons sought medical care. Care seeking was less frequent among adults and when there had already been another case within the household.

Epidemiologic surveillance systems and studies of influenza are typically based on cases of medically diagnosed ILI. Therefore, the proportion of persons not seeking medical care is usually unknown and not taken into account. Ideally, epidemiologic surveillance should consider these persons to avoid underestimating the actual magnitude of the disease (*7*). These persons may increase spread of the disease (*8*) and may also be more likely to self-medicate, resulting in possible risks to their health (*9**,**10*). Persons who do not seek medical care do not contribute to the cost of influenza from the health system perspective, but they do from the point of view of society because they can result in lost work hours, more medications used, and increased need for care (*11*).

When influenza symptoms were present, adults and adolescents sought medical care less often than children. This finding may explain in part why the incidence of medically diagnosed ILI is usually much higher among children (*4**,**6**,**12*).

This study was conducted during the pandemic influenza (H1N1) 2009 season. Therefore, its results may not be generalizable to other influenza seasons (*4*). Nevertheless, the study was conducted when pandemic (H1N1) 2009 virus had already been circulating in the population for several months, the initial alarm had abated, and the level of medical care had returned to levels similar to that for seasonal influenza.

We included symptoms of influenza that were reported by concerned persons or concerned parents. Thus, persons with mild symptoms may be underrepresented in our analysis. The probability of making telephone contact may have been higher in households with more members. However, only 8% (19/252) of households were not contacted. Families in whom none of the members had sought medical care were not included in the study, which may overestimate the proportion of persons who sought medical care.

Persons with influenza who did not seek medical care should be taken into account in estimations of the actual incidence of influenza and its effect on the general population. These persons may have a major effect on transmission and should be considered in planning prevention and control measures and in evaluations of the effects of this disease.
